# Effect of different levels of *Lepidium sativum* L. on growth performance, carcass characteristics, hematology and serum biochemical parameters of broilers

**DOI:** 10.1186/s40064-016-3118-0

**Published:** 2016-08-30

**Authors:** Kassa Shawle, Mengistu Urge, Getachew Animut

**Affiliations:** 1 Department of Animal and Range Sciences, Wolaita Sodo University, P.O. Box 138, Wolaita Sodo, Ethiopia; 2 School of Animal and Range Sciences, Haramaya University, P.O. Box 138, Dire Dawa, Ethiopia; 3 Agricultural Transformation Agency, P.O. Box 708, Addis Ababa, Ethiopia

**Keywords:** Antibiotics, Feed additives, Garden cress, Herb, Phytogenic, Serum biochemical

## Abstract

**Background:**

Frequent use of antibiotics to stimulate growth and health of animals leads to the development of antibiotic-resistant populations of microorganisms. In this context, alternatives like herbs and spices to antibiotics are of importance, as they are natural products. Therefore, the present experiment was designed to evaluate the performance, carcass traits, hematology and serum biochemical parameters of broilers fed ration containing varying levels of *Lepidium satvium* (Garden cress) seed powder as feed additive up to the age of 42 days.

**Methods:**

A total of 204 Cobb-500 day-old broiler chicks were randomly distributed into four treatments with three replicates of 17 chicks each. Garden cress (GC) was included in the ration at 0 (0GC), 0.75 (0.75GC), 1.50 (1.5GC) and 2.25 % (2.25GC) in each treatments. Growth performance, carcass characteristics, heamatology, serum biochemical and economic efficiency parameters were observed.

**Results:**

The crude protein, ether extract and crude fiber content of GC were 22.4, 25.7 and 10.5 %, respectively. Daily dry matter intake and average daily gain during the entire experimental period were affected (p < 0.01) by the treatment diet. Dry matter intake increased with increasing GC level in the ration. Average daily gain for the entire period was greater (p < 0.005) for 0.75 and 1.5GC than 0 and 2.25GC. Groups consumed GC containing ration had better (p < 0.004) drumstick percentage. However, most carcass parts and giblet weight were not affected (p > 0.05). Sex differences were significant for eviscerated, carcass and kidney percentages with greater values for females than males. All hematological parameters were within the normal range. However, inclusion of GC improved (p < 0.05) hemoglobin, packed cell volume and red blood cell count. Group consumed GC containing ration exhibited significant decrease in serum glucose, triglycerides and cholesterol concentration.

**Conclusion:**

Inclusion of GC up to 2.25 % result in no any adverse effect on the health of broilers. Based on the production parameters used in the study, GC can be included as feed additive at a level of 0.75 % in the total ration for better and positive results on biological performance and health status of broilers.

## Background

Animal feed additives are used worldwide for many different reasons. Some are included in animals’ diet to cover the needs of essential nutrients and others to improve health of the animals, feed intake, growth performance and therefore optimize feed utilization. Current trend in animal nutrition is towards feeding of more “natural” diets and the use of some additives like anti-microbial agents in animal feed are not legally allowed in many countries in the world. This has created an increased interest in the feed industry to look for other alternatives, which could be accepted by the consumers. Recently, probiotics, prebiotics, herbs, spices or botanicals (e.g., essential oils), enzymes and minerals are being considered as good options (Patterson and Barkholder 2003).

Herbs, spices and their extracts can be relevant in many different ways for the production of healthy chickens. They provide antioxidants and antimicrobial property (Cross et al. 2007), help in immunity development and promote growth (Ko et al. 2008). Herbs and botanicals contain many different antioxidants with a high potential for the protection of nutrients against oxidation in the digestive tract, in the process of metabolism as well as in the products (Wenk 2003). They can regulate feed intake and stimulate digestive secretions, result in optimized digestion capacity and reduced risk of digestive disorders (Bunyapraphatsara 2007).

Some of the medical effects of herbs and spices are related to their secondary metabolites such as phenols, essential oils, and saponins (Tipu et al. 2006). Many herbs have a long history of use, even prehistoric use, in preventing or treating human and animal diseases, because of their availability, easy usage and non-side effects. Nevertheless, research results on the use of herbal mixtures in broiler diets have produced inconsistent results. Some authors declared positive effects on broiler performance (Cross et al. 2007), while others noted no influence on body weight gain, feed intake or feed conversion (Ocak et al. 2008).

*Lepidium sativum* (commonly known as garden cress) is long been used by farmers for disease treatment and human diets in Ethiopia (Mammo 2006). Garden cress leave, roots and seeds are considered as one of the popular medicinal herbs used in many countries as a good mediator for bone fracture healing (Wadhwa et al. 2012). The seed extract and leaves of the plant also are traditionally used to control many clinical problems such as anti-asthmatic, antiscorbutic, aperient, diuretic, galactogogue, poultice, stimulant and control of blood pressure (Maghrani et al. 2005). As an attribute to its high content of essential fatty acids (Oleic, 30.6 % and linolenic, 29.3 %) and high fat percent (18–24), large concentration of tocopherols, good amount of lignans and antioxidants, which can stabilize the n-3 polyunsaturated fatty acids in its seed oil, garden cress seeds are known to increase weight gain (Diwakar et al. 2008). This herb also is known to improve nutrient digestibility (Mellor 2000). These beneficial properties of the plant is hypothesized to occur if used as feed additive in broiler ration; and may improve growth, feed conversion ratio and quality of carcass, stimulate immune system, and reduce the assumed health risk associated with the consumption of animal products grown on diet containing synthetic or anti-microbial agents. Therefore, the current study was conducted with the objective to evaluate the effect of using different levels of garden cress on performance, carcass characteristics, hematology, blood biochemical and economic efficiency and to determine the safety level of its inclusion in broiler ration.

## Methods

### Experiment site

The experiment was conducted at Haramaya University poultry farm, which is located 515 km east of the capital, Addis Ababa. The site is situated at an altitude of 1980 mater above sea level, 9° 26′ N latitude and 42° 3′ E longitude. The mean annual rainfall of the area is 780 mm and the average minimum and maximum temperatures are 8 and 24 °C, respectively (Samuel 2008).

### Feed ingredients and ration formulation

The feed ingredients used to formulate the experimental rations for the study are presented in Table 1. After contaminant materials were removed the GC was hammer milled to powder. Corn grain, noug seed (*Guizotia abyssinica*) meal and limestone were milled through a sieve size of 5 mm. Chemical composition of the major feed ingredients was determined from representative samples. Based on the chemical analysis result, four isocaloric and isonitrogenous treatment rations containing GC at levels of 0 % (0GC), 0.75 % (0.75GC), 1.5 % (1.5GC), and 2.25 % (2.25GC) of the total ration were formulated by using Feedwin interactive software. Birds were fed according to the recommendations described by Leeson and Summers (2005).

**Table 1:** Ingredients (%) and chemical composition (% DM basis) of different rations

Parameter	Treatments							
	0GC		0.75GC		1.5GC		2.25GC	
	Starter	Finisher	Starter	Finisher	Starter	Finisher	Starter	Finisher
Ingredient composition (%)								
Corn grain	37.3	49.0	37.5	49.0	37.0	48.0	36.0	48.0
Wheat middling	16.0	12.0	15.0	11.5	14.6	12.0	16.0	11.0
Noug seed meal	19.0	16.2	19.0	16.0	19.0	15.7	19.0	16.0
Soybean meal	25.0	20.0	25.0	20.0	25.0	20.0	24.0	20.0
Garden cress	0.00	0.00	0.75	0.75	1.50	1.50	2.25	2.25
Premixa^a^	0.75	0.75	0.75	0.75	0.75	0.75	0.75	0.75
Salt	0.40	0.40	0.40	0.40	0.40	0.40	0.40	0.40
Limestone	1.30	1.40	1.30	1.40	1.30	1.40	1.30	1.40
dl-meth.	0.10	0.10	0.10	0.10	0.10	0.10	0.10	0.10
l-Lysine HCL	0.20	0.15	0.20	0.15	0.20	0.15	0.20	0.15
Chemical composition (% DM basis)								
Dry matter	93.0	92.8	93.0	92.8	93.1	92.9	93.0	92.9
Crude protein	23.1	20.2	23.1	20.2	23.2	20.2	23.0	20.3
Ether extract	4.62	4.51	4.78	4.67	4.94	4.82	5.10	5.00
Crude fibre	6.91	6.52	7.09	6.52	7.13	6.56	7.11	6.61
ME (kcal/kg DM)	3123	3203	3130	3212	3135	3218	3138	3221
Calcium	0.94	0.96	0.95	0.96	0.95	0.96	0.95	0.97
Phosphorus	0.51	0.51	0.53	0.51	0.52	0.53	0.54	0.53

*DM* dry matter, *ME* metabolizable energy, *GC* garden cress, *0GC* ration containing 0 % GC; *0.75GC* 0.75 % GC; *1.5GC* 1.5 % GC,* 2.25GC* 2.25 % GC

^a^Premix = 25 kg contains: Vitamins: Vit. A (E672), 75,000,000 IU; Vit. D3 (E671), 25,000,000 IU; Vit. E (all-rac-alpha tocopherylacetate) (3a700), 20,000 mg; Vit. K3, 2000 mg; Vit. B1, 1500 mg; Vit. B2 (riboflavin), 5000 mg; Vit. B3 (calcium-D-pantothenate), 9001 mg; Vit. B6 (3a831), 5000 mg; Vit. B12 (cyanocobalamin), 25,000 mcg; Vit. pp (nicotinic acid), 30,003 mg; Folic Acid, 1000 mg; Biotin, 100,000 mcg, Choline, 648,750 mg; Minerals: Iron, 45,000 mg; Copper (Cu, E4), 15,000 mg; Manganese (Mn,E5), 75,001 mg; Zinc oxide-Zinc (Zn,E6), 70,001 mg; Iodine (I,E2), 2000 mg; Selenium (Se, E8), 400,050 mcg; Calcium, 1,231,662 mg; Magnesium, 12,687 mg; Sodium, 952 mg; Chloride, 185,313 mg; BHT, 500 mg

### Managements of experimental chickens

The experimental house and pens, watering and feeding troughs were thoroughly cleaned, disinfected and sprayed against external parasites before placing the chicks. Two hundred and four unsexed day old Cobb-500 broiler chicks with initial body weight of 45 ± 1.4 g (mean ± SD) were randomly divided into four dietary treatments each with three replication of 17 chicks. The birds were vaccinated with HB1 at day 7 and Lasota booster dose at day 21 as eye drop and in drinking water, respectively as a preventive treatment against Newcastle disease. The chicks were brooded using 250 W infrared electric bulbs with gradual height adjustment as sources of heat and light. Grass hay was used as a litter material with a depth of approximately 7 cm. Feed and clean tap water were offered ad libitum throughout the experiment.

## Measurements

### Intake and body weight gain

The experimental period lasted for a total of 42 days (21 days for starter and 21 days for finisher phase). The amount of feed offered and leftover was recorded daily and the amount consumed was determined as the difference between the feed offered and leftover. Broilers were weighed weekly in a group per pen and pen average was calculated. Body weight (BW) change was calculated as the difference between the final and initial BW. Average daily gain (ADG) was calculated as BW change divided by the number of experimental days. Dry matter conversion ratio was computed as the ratio of daily DM consumption to ADG. Health status of the birds was monitored throughout the experiment and when occurred, cause of mortality was investigated by a veterinarian and a total death was expressed as percent of broilers housed.

### Carcass measures

At the end of the experiment, four randomly selected birds’(two males and two females) with body weight close to the average of the group were taken from each replicate, starved for 12 h, weighed immediately before slaughter, exsanguinated by severing the neck, and dry de-feathered by hand plucking. Birds were eviscerated and carcass cuts and non-edible offal components were determined according to the procedure described by Kekeocha (1985). Dressed weight was measured after the removal of blood and feather and the dressing percentage was calculated as the proportion of dressed carcass weight to slaughter weight. Eviscerated carcass weight was determined after removing blood, feather, shank, head, kidney, lungs, pancreas, crop, proventriculus, small intestine, large intestine, caeca and urogenital tracts. The eviscerated percentage was determined as the proportion of slaughter weight. From eviscerated carcass weight drumstick, thigh, breast meat, heart, gizzard and liver were separated, weighed and calculated as a percentage of slaughter weight. Fat around the proventriculus, gizzard, against the abdominal wall and the cloacae were separated, weighed and expressed as a percentage of slaughter weight.

### Hematology

Blood samples were collected from the jugular vein of 3 broilers (per replicate) used for carcass evaluation into a 5 mL sterile syringe using a 23-gauge needle. Each blood sample was immediately transferred into a tube containing ethylene diamine tetra-acetic acid (EDTA) as an anticoagulant. The red blood cell (RBC) and white blood cell (WBC) were counted using haemocytometer (Irizaary-Rovira 2004). The hemoglobin (Hb) concentration was determined by matching acid hematin solution against a standard colored solution found in Sahlis hemoglobinometer. Packed cell volume (PCV) was measured by microhaematocrit method. Mean corpuscular volume (MCV), mean corpuscular hemoglobin concentration (MCHC) and mean corpuscular hemoglobin (MCH) were computed as described by Irizaary-Rovira (2004).

### Serum biochemical parameters

Blood was collected from the same broilers from which sample for hematological analysis was taken. A plain tube was used to collect the blood samples. Serum was separated after centrifugation at 3000 rpm×*g* for 15 min and stored at −20 °C until used. Serum alanine aminotransferase (ALT), aspartate aminotransferase (AST), alkaline phosphatase (ALP) activities, cholesterol, glucose and triglycerides concentrations were measured by using enzyme/buffer and substrate kits according to the Standard Operating Procedure of Harari Health Research and Regional Laboratory, Harar, Ethiopia. Total serum protein was determined by refractometer (George 2001). Total serum immunoglobulin concentration was determined by serum zinc sulfate turbidity test by reading the optical density of the test and the control separately at 545 nm by using spectrophotometer (Mondesire 2004).

### Chemical composition of feed

Representative samples of the feed ingredients and rations were analyzed for dry matter (DM), crude fiber (CF), total ash (TA), ether extract (EE), and Kjeldahl nitrogen (N) (AOAC 1990). The CP content was determined by multiplying N by 6.25. Calcium and total phosphorus content were determined by atomic absorption methods (AOAC 1990). Metabolizable energy (ME) content of the experimental diets was determined as:

$${\text{ME}}\;\left( {{\text{kcal}}/{\text{kg}}\;{\text{DM}}} \right) = 3951 + 54.4{\text{EE}} - 88.7{\text{CF}} -40.8\;{\text{ash}}.$$

### Production and/or economic efficiency

Economic efficacy of feeding ration containing different levels of garden cress seed powder was evaluated by calculating European Broiler Index (EBI) and Production Efficiency Factor (PEF) (Marti et al. 2007; Marcu et al. 2013).

$${\text{EBI}} = \frac{{{\text{Viability }}\left( {\text{\%}} \right) \times {\text{ADG }}\left( {{\text{g}}/{\text{chick}}/{\text{d}}} \right)}}{{{\text{FCR }}\left( {{\text{kg}}\;{\text{feed}}/{\text{kg}}\;{\text{gain}}} \right) \times 10}}$$

$${\text{PEF}} = \frac{{{\text{viability}}\;(\% ) \times {\text{BW}}\;({\text{kg}})}}{{{\text{Age}}\;(d) \times {\text{FCR}}\left( {{\text{kg}}\;{\text{feed}}/{\text{kg}}\;{\text{gain}}} \right)}} \times 100$$

where viability (%) = chicks alive at the end of the period (%), age (day) = the age of the chick at slaughter, BW = final life body weight of the broilers, ADG = average daily gain, d = day, FCR = feed conversion ratio.

The economic benefit also was estimated by partial budget analysis (Upton 1979). Variable cost was determined as the difference between feed expense to formulate each treatment diet and prices of carcass. The net income (NI) was calculated as the amount of money which is left when total variable cost (TVC) was subtracted from total returns. Marginal rate of return (MRR) was calculated by dividing net income to variable cost.

### Statistical analysis

Data were analyzed using the general linear model procedures of Statistical Analysis Systems software (SAS Institute 2008), with the model containing treatments for performance, hematological and serum biochemical parameters and treatment and sex for carcass characteristics. Differences between treatment means were separated using Duncan’s multiple range test (p < 0.05).

## Results

### Chemical composition of feed ingredients and the ration

As expected, the four treatment rations were nearly isocaloric and isonitrogenous (Table 1). Nutrient concentration of ingredients used for the formulation of the experiment ration was similar to results reported in earlier studies for the same feed (Etalem et al. 2013). Fiber concentration in noug seed meal (NSM) (18.5 %) and garden cress (10.5 %) was higher as compared to the other ingredients. Garden cress consist high percentages of ether extract (EE) (25.7 %) and ME (4158 kcal/kg DM) than the other ingredients.

### Intake and body weight gain

Daily DM intake, final body weight and average daily body weight gain (ADG) during the starter phase (Table 2) did not differ among birds fed ration containing variable levels of garden cress (GC). However, the best (p < 0.000) feed conversion ratio (FCR) were obtained from birds fed with 0.75GC followed by the control (0GC) ration. Feed consumption during the finisher phase increased (p < 0.05) with increasing level of GC in the ration. Higher (p < 0.028) ADG in finisher phase was observed for 0.75GC than 0GC and 2.25GC rations. The FCR of broilers fed with 0GC, 0.75GC and 1.5GC rations during this period was not different from each other, but were better (p < 0.024) than those fed with 2.25GC. Results for the entire experimental period showed that broilers consumed 0.75GC and 1.5GC rations resulted in superior (p < 0.005) ADG than those fed with 0GC and 2.25GC rations. Only two mortalities occurred at the early stage of the experiment, which could be attributed to transport stress and vaccine activation. As a result, there was no significant difference in mortality rate among treatments.

**Table 2:** Performance of broilers fed ration containing different levels of Garden cress seed powder

Parameters	Treatments				SEM	p value
	0GC	0.75GC	1.5GC	2.25GC		
1–21 days (starter phase)						
IBW (g)	44.9	44.4	45.2	45.5	0.91	0.867
DMI (g/bird/day)	46.7	46.5	49.3	48.6	1.01	0.205
Total feed (g/bird)	980	976	1035	1021	21.1	0.205
FBW (g)	494	512	500	486	10.0	0.356
BWG (g/bird)	449	468	455	440	10.1	0.340
ADG (g)	21.4	22.3	21.6	21.0	0.48	0.340
FCR	2.18^b^	2.08^c^	2.28^a^	2.32^a^	0.02	0.000
Mortality (%)	0.00	5.88	5.88	0.00	–	–
22–42 days (finisher phase)						
DMI (g/bird/day)	125^c^	130^b^	134^ab^	135^a^	1.28	0.001
Total feed (g/bird)	2623^c^	2730^b^	2812^ab^	2842^a^	26.9	0.001
IBW(g)	494	512	500	486	10.0	0.356
BWG (g/bird)	1078^bc^	1146^a^	1134^ab^	1066^c^	17.6	0.028
ADG (g)	51.3^bc^	54.6^a^	54.0^ab^	50.8^c^	0.84	0.028
FCR	2.44^b^	2.38^b^	2.48^b^	2.67^a^	0.05	0.024
Mortality (%)	0.00	0.00	0.00	0.00	–	–
1–42 days (entire experiment)						
DMI (g/bird)	85.8^c^	88.3^bc^	91.6^ab^	92.0^a^	1.08	0.010
Total feed (g/bird)	3604^c^	3707^bc^	3847^ab^	3863^a^	45.2	0.010
FBW (g)	1572^b^	1658^a^	1634^a^	1551^b^	16.9	0.006
BWG (g/bird)	1527^b^	1614^a^	1589^a^	1506^b^	16.7	0.005
ADG (g)	36.4^b^	38.4^a^	37.8^a^	35.9^b^	0.40	0.005
FCR	2.36^b^	2.30^b^	2.42^b^	2.57^a^	0.04	0.006
Mortality (%)	0.00	5.88	5.88	0.00	–	–

*SEM* standard error of the mean, *ADG* average daily gain, *BWG* body weight gain, *IBW* initial body weight, *FBW* final body weight, *FCR* feed conversion ratio, *DMI* dry matter intake, *GC* garden cress, *0GC* ration containing 0 % GC, *0.75GC* 0.75 % GC; *1.5GC* 1.5 % GC, *2.25GC* 2.25 % GC

^abc^Means in a row with different superscripts differ (p < 0.05)

### Carcass measures

The slaughter weight, dressed weight, eviscerated weight, eviscerated percentage, carcass weight, and heart, liver, gizzard and kidney percentages were not significantly different among treatments (Table 3). However, breast, thigh, drumstick, thigh + drumstick and abdominal fat percentages were significantly affected by treatments. Higher breast and thigh percentage were exhibited in broilers consumed 0.75GC than 2.25GC but the former is not different from those consumed 0GC and 1.5GC rations. Lower drumstick percentage was recorded for birds fed with 0GC than other rations. Higher abdominal fat percentage was recorded in broilers consumed 0.75GC than 0GC and 1.5GC rations. Sex significantly affected eviscerated, carcass and kidney percentage with greater values for females than males. The rest of the parameters were not different (p > 0.05) between the sexes.

**Table 3:** Carcass components of broilers fed ration containing different levels of garden cress seed powder

Parameters	Treatments						Sex			
	0GC	0.75GC	1.5GC	2.25GC	SEM	p value	Male	Female	SEM	p value
Slaughter wt (g)	1615	1640	1588	1545	46.5	0.569	1643	1551	32.9	0.053
Dressed wt (g)	1481	1504	1460	1413	43.4	0.542	1506	1424	30.8	0.068
Dressing %	91.8	91.7	92.0	91.4	0.31	0.584	91.6	91.8	0.22	0.501
Eviscerated wt (g)	1203	1233	1178	1141	35.0	0.328	1212	1165	24.8	0.197
Eviscerated %	74.5	75.2	74.3	73.9	0.52	0.444	73.8^b^	75.1^a^	0.37	0.015
Carcass %	69.6	70.5	69.6	68.9	0.55	0.295	69.0^b^	70.3^a^	0.39	0.031
Breast %	25.6^ab^	27.0^a^	26.3^a^	24.2^b^	0.67	0.032	25.3	26.3	0.47	0.149
Thigh %	10.3^ab^	10.7^a^	10.1^ab^	9.93^b^	0.17	0.027	10.2	10.3	0.12	0.380
Drumstick %	8.28^b^	8.87^a^	9.07^a^	8.93^a^	0.15	0.004	8.84	8.74	0.11	0.502
T + D %	18.5^b^	19.5^a^	19.2^ab^	18.9^ab^	0.26	0.062	19.0	19.1	0.19	0.868
Gizzard %	2.12	2.07	1.95	2.21	0.10	0.359	2.06	2.11	0.07	0.656
Liver %	2.17	1.97	2.08	2.09	0.08	0.402	2.09	2.07	0.06	0.886
Heart %	0.64	0.61	0.62	0.66	0.04	0.790	0.62	0.65	0.03	0.361
Kidney %	0.65	0.68	0.64	0.64	0.04	0.933	0.60^b^	0.71^a^	0.03	0.010
AF %	2.12^b^	2.72^a^	2.00^b^	2.25^ab^	0.16	0.021	2.22	2.33	0.12	0.750

*SEM* standard error of the mean, *GC* garden cress, *0GC* ration containing 0 % GC, *0.75GC* 0.75 % GC, *1.5GC* 1.5 % GC, *2.25GC* 2.25 % GC, *AF* abdominal fat; *T* *+* *D* thigh + drumstick, *wt* weight

^ab^Means in a row and under treatment or sex with different superscripts differ (p < 0.05)

### Hematology and serum biochemical

With the exception of mean corpuscular hemoglobin concentration percentage (MCHC  %), all hematological parameters were significantly affected (p < 0.05) by GC inclusion in the ration of broilers (Table 4). Generally, values for hemoglobin (Hb), packed cell volume (PCV) and red blood cell (RBC) count were higher at least numerically, in groups consumed ration containing GC. On the other hand, the values of mean corpuscular volume (MCV), mean corpuscular hemoglobin (MCH) and white blood cell (WBC) counts were greater in birds consumed 0GC ration than 0.75GC, 1.5GC and 2.25GC.

**Table 4:** Hematology of broilers fed different levels of garden cress seed powder

Parameters	Treatments				SEM	p value
	0GC	0.75GC	1.5GC	2.25GC		
Hb (g/100 mL)	8.38^c^	9.67^a^	8.89^bc^	9.28^ab^	0.21	0.002
PCV (%)	27.6^b^	30.4^a^	29.4^ab^	29.2^ab^	0.65	0.045
RBC (10^6^cells/mm^3^)	2.56^c^	3.17^a^	3.10^a^	2.88^b^	0.08	0.000
MCV (fL)	108^a^	95.9^c^	94.7^c^	103^b^	1.71	0.000
MCH (pg)	33.0^a^	30.5^bc^	29.1^c^	32.3^ab^	0.71	0.003
MCHC (%)	30.4	31.8	30.8	31.5	0.52	0.221
WBC (10^3^cells/mm^3^)	18.0^a^	17.1^ab^	15.1^b^	16.1^ab^	0.68	0.032

*SEM* standard error of the mean, *Hb* hemoglobin, *PCV* packed cell volume, *RBC* red blood cell, *MCV* mean corpuscular volume, *MCH* mean corpuscular hemoglobin, *MCHC* mean corpuscular hemoglobin concentration, *WBC* white blood cell, *GC* garden cress; *0GC* Ration containing 0 % GC, 0.*75GC* 0.75 % GC, *1.5GC* 1.5 % GC, *2.25GC* 2.25 % GC

^abc^Means in a row with different superscripts differ (p < 0.05)

Except alanine aminotransferase (ALT), total serum protein (TSP) and immunoglobulin, all other blood biochemical parameters were significantly (p < 0.05) affected by the different levels of GC inclusion in the ration (Table 5). Birds fed 0.75GC and 1.5GC rations had higher aspartate aminotransferase (AST) activities than those fed 0GC and 2.25GC. Although the trend in concentration of alkaline phosphatase (ALP) activity was not consistent, broilers consumed 0GC and 1.5GC ration displayed higher ALP activity than 0.75GC. Broilers consumed ration containing GC exhibited a decrease in serum glucose, triglyceride and cholesterol in comparison with 0GC group.

**Table 5:** Serum biochemical parameters of broilers fed different levels of garden cress powder

Parameters	Treatments				SEM	p value
	0GC	0.75GC	1.5GC	2.25GC		
AST (IU/L)	202^bc^	219^ab^	227^a^	193^c^	7.09	0.008
ALT (IU/L)	4.11	4.22	4.22	4.22	0.41	0.996
ALP (IU/L)	5213^a^	3724^b^	5055^a^	4416^ab^	388	0.050
Glucose (mg/dL)	204^a^	183^b^	180^b^	178^b^	6.43	0.024
Triglycerides (mg/dL)	35.7^a^	29.9^ab^	29.0^ab^	24.7^b^	2.21	0.013
Cholesterol (mg/dL)	125^a^	101^c^	111^bc^	113^b^	3.65	0.000
Total protein (g/dL)	3.04	3.07	2.87	2.98	0.38	0.769
Immunoglobulin(mg/dL)	1.44	1.31	1.36	1.34	0.15	0.934

*AST* aspartate aminotransferase, *ALT* alanine aminotransferase, *ALP* alkaline phosphatase, *SEM* standard error of the mean, *GC* garden cress, *0GC* ration containing 0 % GC, *0.75GC* 0.75 % GC, *1.5GC* 1.5 % GC, *2.25GC* 2.25 % GC

^abc^Means in a row with different superscripts differ (p < 0.05)

### Production efficiency and economic return

Production efficiency factor (PEF) and European broiler index (EBI) values during the starter phase were higher in broilers fed with 0.75GC ration as compared to those consumed 1.5GC and 2.25GC, but the control did not differ from all other treatments (Table 6). During the finisher phase, EBI was higher in birds consumed 0.75GC and 1.5GC than 2.25GC, but was similar with 0GC group. The PEF value during the entire rearing period was also higher (p < 0.006) for 0.75GC group followed by 1.5GC than broilers fed with the control and 2.25GC rations.

**Table 6:** Production efficiency and profitability of broiler ration containing different levels of garden cress

Description	Treatments					
	0GC	0.75GC	1.5GC	2.25GC	SEM	p value
Production efficiency						
Starter PEF	108^ab^	115^a^	103^b^	99.7^b^	3.13	0.041
Starter EBI	98.1^ab^	105^a^	93.2^b^	90.4^b^	2.97	0.041
Finisher EBI	211^ab^	229^a^	218^a^	190^b^	7.74	0.040
Entire period PEF	159^b^	172^a^	161^ab^	144^c^	3.85	0.006
Entire period EBI	154^ab^	164^a^	153^ab^	139^b^	4.36	0.027
Profitability/partial budget						
Cost (Birr)						
Day old chick cost (Birr)	20.0	20.0	20.0	20.0		
Total feed consumed/bird (kg)	3.60	3.71	3.85	3.86		
Per unit feed cost (Birr)	6.93	7.12	7.30	7.48		
Total feed cost (birr/bird)	25.1	26.4	28.1	28.9		
Total cost (Birr)	44.9	46.4	48.1	48.9		
Revenue						
Average carcass weight (kg)	1.11	1.18	1.10	1.06		
Price/kg of carcass (supermarket)	80.0	80.0	80.0	80.0		
Total return (Birr)	88.8	94.4	88.0	84.8		
Net return/bird (birr)	43.9	48.0	39.9	35.9		
Marginal rate of return	0.98	1.03	0.83	0.74		

*GC* garden cress, *OGC* ration containing 0 % GC, *0.75GC* 0.75 % GC, *1.5GC* 1.5 % GC, *2.25GC* 2.25 % GC, *EBI* European broiler index, *PEF* production efficiency factor

^abc^Means in a row with different superscripts differ (p < 0.05); 1 Birr = 0.05US$

The highest net profit was obtained from the sale of meat obtained from broilers fed with ration containing 0.75GC followed by 0GC and 1.5GC rations (Table 6). The least profit was earned from group consumed 2.25GC ration. The marginal rate of return due to the inclusion of GC in the diet of the chicken was 1.03 Birr for 0.75GC, but it was less than one for 1.5GC and 2.25GC groups indicating lose in profitability at higher level of GC inclusion. The overall result of production efficiency measure and partial budget indicate that feeding ration containing lower level of GC provided the highest net benefits per kg of body weight gain.

## Discussion

Lipid, protein and moisture content of GC used in the present study were comparable with previously reported values of 28.0, 24.2 and 3.9 respectively (Zia-Ul-Haq et al. 2012). However, ash and crude fiber concentration were higher than the values reported by the same authors. Sharma and Agarwal (2011) reported comparable composition of CP (23.5 %), ash (5.70 %), phosphorous (1.65 %) and calcium (0.31 %), but lower level of fat (15.9 %) for GC. The variability in the composition of GC reported in different experiments could be an attribute of differences in plant variety, agronomic practices, stage of collection of seeds and climatic and geological condition of area from where seeds are collected. The higher ash contents of GC seed in the present study indicate that it is an appreciable source of minerals. The low moisture content of GC is also an index of stability and increased shelf life of seeds (Marangoni and Alli 1988). The higher lipid contents of GC indicate that the seeds are rich in energy.

Inclusion of GC in broiler ration appeared to increase feed intake during the later stage of development. The increased feed intake with increasing level of GC may indicate its positive influence on appetite of the birds and nutrient digestion. Similar to the present result, earlier works showed increased intake (Windisch et al. 2007) as well as digestion (Mellor 2000) in birds consumed feeds prepared with inclusion of herbs and various plant extracts due to their appetite and digestion stimulating properties. Herbs and their extracts are known to contribute to the nutrient requirements of the animals (Frankic et al. 2009) due to their complex mixture of bio-active components and some unidentified factors and their stimulating effect of digestive enzymes (Jang et al. 2007). Nevertheless, the increased intake in the present experiment did not improve average daily gain at the higher level of inclusion, may be due to the presence of certain anti-nutritional factors. According to Agarwal and Sharma (2013), whole GC seed flour contain tannins, phytic acids, oxalic acid and cyanogens (51, 0.77, 136 and 5.50 mg/100 g, respectively), which might have hampered the bioavailability of nutrients. Similar to the present study, moderate level of GC inclusion in rats ration (Al Hamedan 2010) resulted in higher weight gain and feed efficiency ratio than the control group. Likewise, positive effect of GC on live weight, FCR and egg production were reported (Nada 1999) when included at low level in the ration of hen. Feeding GC seeds to rat in water suspension did not improve body weight in the first 3 weeks, but body weight significantly decreased after 6 weeks at doses of 4 g/100 mL and 8 g/100 mL compared to 2 g/100 mL and the control groups indicating high dose negatively influenced body weight gain (Bafeel and Ali 2009).

Improved carcass cut at 0.75 and 1.5 % level of GC might be related to high concentration of essential compounds such as linolenic fatty acids and flavonoids in the GC seeds. According to DeLany et al. (1999) high level of conjugated linoleic acid improve the ratio of lean body mass to fat, especially in the abdomen, and enhances muscle growth. Flavonoids also prevent oxidation of lipid and allow high amounts of lean cut to accumulate in the organs such as breast and improve the weights and proportions of the organs. Inclusion of GC did not affect the weight of gizzard, liver and kidney. According to Bone (1979) abnormalities in the weights of the internal organs arise because of increased metabolic rate of the organs in an attempt to reduce toxic elements or anti-nutritional factors to non-toxic metabolites. Thus, the absence of treatment effect on these organs implies that the test diets did not contain appreciable toxin at levels used in the present study to impair the development of the organs. Higher eviscerated percentage in females than males in the current study was in agreement with that reported by Etalem et al. (2013). Previous studies also noted females to have greater proportion of breast meat than males (Rondelli et al. 2003), which was not the case in the current study. However, there are also studies that reported males to have higher proportion of thigh and drumstick than females (Etalem et al. 2013), which is not observed in our experiment.

In general, the values of hematological indices fall within safety limit of healthy broiler chickens (Islam et al. 2004; Wakenell 2010). Normal hematological values portray the nutritional status of the animal. Thus, the normal values observed in the present study indicate the adequacy of nutrients for the birds. It also implies that the immune systems of the birds are adequate. Even though Hb, PCV and RBC values are within the normal range, the higher values observed in broilers consumed ration containing GC as compared to the control diet suggest that GC improved nutrient utilization and assimilation into the blood stream for use by the birds and enhanced blood formation due to availability of essential nutrients and better feed intake. When the level of GC increased, Hb concentration also was increased. This might be due to the presence of significant mineral elements especially Fe, Mn, Cu and Ca in the GC powder (Zia-Ul-Haq et al. 2012).

The value of cholesterol obtained in the present study is similar to that reported earlier (Campbell 2012). Nevertheless, lower values of glucose, triglycerides and cholesterol in group consumed diet containing GC as compared to those fed diet without GC could be an indication of its hypocholestrolemic properties. This finding agreed with the result reported by Amawi and Aljamal (2012) who observed decreased glucose, cholesterol and triglycerides and low density lipoprotein in hypercolestrolemic and diabetic rats after the administration of GC extract (20 mg/kg) for 4 weeks. Likewise, research result revealed that the level of serum cholesterol, triglycerides, LDL-c and VLDL-c levels decreased when hypercholesterolemic rats were treated daily with either drug (Gemfiibrazil capsule 9 mg), GC extract (5 %) or GC powder (10 %) (Al Hamedan 2010). Youssef et al. (2014) also confirmed serum cholesterol, triglycerides and LDL-c lowering properties of GC oil (25, 50 and 75 %) in albino rats. This could in part be due to the high copper content of GC (Zia-Ul-Haq et al. 2012) as it was known to have a blood cholesterol lowering effect. The other possible reason for the observed decrease in blood sugar level in the GC containing diet is due to the presence of linolenic acids. Bryan et al. (2009) noted that the primary fatty acids in GC oil are oleic (30.6 %) and linolenic acids (29.3 %). Lower serum cholesterol indicates better cardiac function. Hence, broilers raised on diet containing GC may be healthier than those consumed the control diet provided all other factors affecting broilers’ cardiovascular system were normal.

The blood glucose concentration in normal birds ranges from 200 to 500 mg/dL (Campbell 2012). In the present study, the level of blood glucose in the GC groups is a little bit lower than this range. However, the level is not bellow the critical value of 150 mg/dL (Brar et al. 2000) indicating no stress or abnormal glucose level related problem is suspected. Similarly, triglyceride levels of broilers fed GC diets were lower as compared to the control diet. Reduced glucose and triglycerides concentration in groups consumed GC containing ration might be due to the beneficial effects of flavonoids in medicinal plants such as GC. Some flavonoids possess insulin- like properties and thereby are able to reduce blood glucose levels (Brahmachari 2011). The serum protein concentrations in all treatments were not significantly different and found within the normal range (Campbell 2012). The total immunoglobulin concentration also was not affected by the treatment diets. This is in agreement with Mountzouris et al. (2010) who evaluated the effect of different levels of probiotics on plasma immunoglobulins and found absence of significant treatment effect.

The AST value observed in all treatments of this experiment was between 193 and 227 IU/L. Campbell (2012) noted that plasma AST activity is considered high when the value is greater than 275 IU/L. The AST and ALT value in this study indicated that there was no damage to heart or liver due to inclusion of GC (Campbell 2012). Youssef et al. (2014) reported the potential of GC oil in reducing AST and ALT levels in hypercholestromic rats. They reported that GC oil supplemented to hypercholestrolimic albino rats (25, 50 and 75 %) reduced the AST and ALT levels than the positive control. Statistically higher ALP activity was observed in 0GC and 1.5GC rations. ALP in the liver is associated with biliary epithelial cells and canalicular membranes of hepatocytes (Center 2007). A variety of hepatobiliary diseases can result in increased serum ALP activity due to increased enzyme production, solubilization of membranes by the action of bile salts, and release of membrane blebs after cell injury (Fernandez and Kidney 2007). Kogut and Powell (1993) also noted that plasma ALP activity appears to be a sensitive indicator of intestinal diseases, such as coccidial infections in the duodenum, jejunum, and cecum. On the other hand, according to Campbell (2012), since ALP is found in multiple tissues, including bone and intestine, the increase in plasma ALP activity is not the result of leakage of the enzyme from the cell but the result of cellular production. Therefore, the increases in the plasma ALP activity are indicative of skeletal growth, nutritional secondary hyperparathyroidism and healing fractures.

Economic efficiency assessment is positively influenced by performance parameters such as BW, ADG, FCR and viability (Broiler Management Manual Ross-308 2009). Increasing values of the two indices (PEF and EBI) at 0.75GC group show that the performances obtained are better than the other groups. EBI values are lower than PEF, because the ADG calculations exclude the chick’s initial weight. However, the EBI and PEF values obtained in all treatments in this study were lower than that accepted in Europe. The normal growth and viability parameters considered normal in Europe is between 200 and 225 PEF units (Shane, n.d.). The lower PEF values might be related to the health, environmental stress or feed quality variations. The partial budget profitability analysis in the current study indicated that the highest net return (48.0 ETB/broiler) was obtained from broilers consumed 0.75GC. The least net return (35.9 ETB/broiler) was obtained from broilers fed 2.25GC. The difference in the net return among treatments was mainly due to the high cost of GC and low body weight gain of broilers at a higher level of inclusion. The MRR implied that each additional unit of 1 Birr cost increment resulted in 1 Birr and additional 1.03 Birr profit for 0.75GC. Therefore, the results of this study indicated that ration containing 0.75GC is potentially profitable than the higher level of GC inclusion in the ration under the conditions of the present experiment.

## Conclusion

The results of this study show that inclusion of GC up to 2.25 % result in no adverse effect on the health of broilers. However, lower level (0.75 %) garden cress inclusion tend to improve body weight, feed efficiency, hematological and blood biochemical indices. Therefore, we conclude that GC can be included in broilers diet as feed additive at a level of 0.75 % of the total ration.

## Abbreviations

ADG: average daily gain
ALP: alkaline phosphatase
ALT: alanine aminotransferase
AST: aspartate aminotransferase
CP: crude protein
DM: dry matter
EBI: European broiler index
FCR: feed conversion ratio
GC: garden cress
Hb: hemoglobin
MCH: mean corpuscular hemoglobin
MCHC: mean corpuscular hemoglobin concentration
MCV: mean corpuscular volume
ME: metabolizable energy
PCV: packed cell volume
PEF: production efficiency factor
RBC: red blood cells
WBC: white blood cells
